# Tomatine Improves Glucose Metabolism and Mitochondrial Respiration in Insulin-Resistant Hepatocyte Cell Lines AML12 and HepG2 via an AMP-Activated Protein Kinase-Dependent Pathway

**DOI:** 10.3390/cells14050329

**Published:** 2025-02-23

**Authors:** Yu Geon Lee, Donghwan Kim

**Affiliations:** Food Functionality Research Division, Korea Food Research Institute (KFRI), Wanju-gun 55365, Jeonbuk-do, Republic of Korea; ugun2@kfri.re.kr

**Keywords:** tomatine, insulin resistance, hepatocytes, AMPK, mitochondrial respiration

## Abstract

Insulin resistance (IR) disrupts hepatic glucose metabolism and mitochondrial function, which contributes to metabolic disorders. The present study examined the effects of tomatine on glucose metabolism in high-glucose-induced IR hepatocytes and explored its underlying mechanisms using AML12 and HepG2 cell models. The results showed that tomatine did not exhibit cytotoxic effects. Under IR conditions, tomatine dose-dependently improved glucose metabolism by enhancing glucose consumption and restoring the mRNA expression of the glucose transporter Glut2 and gluconeogenesis-related genes (*Pepck* and *G6pase*). Mechanistically, tomatine activated the phosphorylation of AMP-activated protein kinase (AMPK) and upregulated the expression of peroxisome proliferator-activated receptor gamma coactivator 1-alpha (PGC1α), reversing the IR-induced suppression of the AMPK/PGC1α pathway. In addition, tomatine enhanced mitochondrial oxidative function by restoring the oxygen consumption rate, increasing ATP production, and upregulating mitochondrial oxidative phosphorylation complex proteins. Both genetic and pharmacological inhibition of AMPK abolished these beneficial effects, confirming its central role in mediating tomatine’s actions. Overall, our findings suggest that tomatine is a promising therapeutic candidate for enhancing hepatic glucose metabolism and mitochondrial function in IR-associated metabolic disorders through AMPK activation.

## 1. Introduction

Abnormal glucose metabolism is a key indicator of insulin resistance (IR), which is a condition that significantly contributes to the prevalence of various metabolic disorders, including type 2 diabetes mellitus (T2DM) [1]. IR is defined by a reduced cellular response to insulin, serving as a key contributor to the development of the metabolic syndrome, obesity, and cardiovascular diseases. In individuals with T2DM or obesity, impaired insulin function prevents glucose from efficiently entering the cells [2]. This leads to the disruption of energy metabolism and blood sugar regulation, resulting in abnormally high blood glucose levels [3]. This disruption arises because the insulin signaling pathway fails to function properly despite elevated blood glucose levels. Moreover, it affects glucose homeostasis, including the activity of the glucose transporter system, and inhibits intracellular glucose uptake [4]. This vicious cycle results in chronic hyperglycemia and its associated complications [2,3,4]. Therefore, understanding the fundamental mechanisms of IR and identifying compounds that positively influence glucose metabolism are important to develop effective treatments and prevention strategies for metabolic diseases.

As the primary functional cells of the liver, hepatocytes play a central role in regulating glucose metabolism and maintaining blood sugar levels through gluconeogenesis and glycogen synthesis [5]. These metabolic processes are crucial for energy homeostasis, particularly during periods of fasting or increased energy demand [6]. The AMP-activated protein kinase (AMPK) pathway is an important regulatory pathway involved in hepatic glucose metabolism, which acts as a metabolic sensor and responds to changes in cellular energy status [7]. When energy levels are low, AMPK is activated to promote catabolic processes that generate ATP by enhancing mitochondrial oxidative phosphorylation (OXPHOS) while inhibiting anabolic processes that consume energy [8]. In the liver, the activation of AMPK suppresses gluconeogenesis, enhances glucose uptake, and promotes fatty acid oxidation, which contribute to restoring energy balance [9]. However, in the context of IR, the activation of AMPK is often impaired despite the hyperglycemic state, which disrupts glucose homeostasis and exacerbates metabolic dysfunction [10]. This suppression of AMPK in IR conditions highlights the complex interplay between metabolic pathways and underscores the need for targeted interventions to restore proper hepatic function [11].

Tomatine, a glycoalkaloid extracted from tomatoes, has shown promising potential in enhancing human health through a variety of bioactive properties, including anti-inflammatory, antioxidant, prebiotic, and cardioprotective effects [12]. Moreover, tomatine supplementation has been reported to reduce serum LDL cholesterol absorption without affecting HDL cholesterol levels [13]. These multifaceted bioactivities make tomatine a promising candidate for the therapeutic management and prevention of chronic diseases, particularly metabolic disorders such as T2DM, obesity, and cardiovascular pathologies [14,15]. However, the precise molecular mechanisms by which tomatine modulates glucose metabolism in IR remain unclear, necessitating further in-depth research.

Therefore, the present study evaluated the effects of tomatine on glucose metabolism disorders in high-glucose-induced IR hepatocytes. Specifically, we determined whether tomatine could improve hepatic glucose availability in the IR state. Furthermore, we investigated the molecular mechanisms underlying the anti-IR effects of tomatine through in vitro cell experiments.

## 2. Materials and Methods

### 2.1. Materials and Reagents

**Figure 1:**
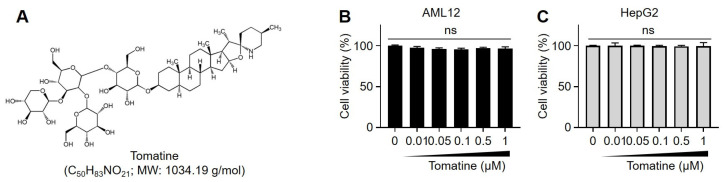
Chemical structure and cytotoxicity of tomatine in hepatocytes. (**A**) Chemical structure of tomatine. (**B**,**C**) AML12 or HepG2 cells were treated with tomatine (0–1 µM) for 24 h, and the cell viability was assessed using WST-1 assay. Data are presented as the mean ± SD (n ≥ 5). Significant differences (*p* < 0.05) were determined using one-way ANOVA followed by Tukey’s post hoc test. “ns” indicates nonsignificant differences.

Tomatine (Purity: 99.90%; Figure 1A) was obtained from MedChemExpress (HY-N2166, Monmouth Junction, NJ, USA). Dimethyl sulfoxide (DMSO; #276855), D-(+)-glucose solution (#G8644), insulin (#I6634), palmitic acid (PA, #P0500), dexamethasone (#D1756), and compound C (#171260, AMPK inhibitor) were purchased from Sigma-Aldrich (St. Louis, MO, USA). Antibodies against phospho(p)-AMPK (Thr 172, #2531; Cell Signaling, Danvers, MA, USA), total(t)-AMPK (#2793; Cell Signaling), peroxisome proliferator-activated receptor gamma coactivator 1-alpha (PGC1α; 66369-1-Ig; Proteintech, Chicago, IL, USA), total OXPHOS cocktail (#ab110413, Abcam, Cambridge, MA, USA), VDAC (#4866; Cell Signaling), and GAPDH (#2118; Cell Signaling) were used for Western blot analysis. The Seahorse XF Cell Mito Stress Test Kit (#103015-100) and Seahorse XF Pro Extracellular Flux Assay Kits (#102418-000) were purchased from Agilent Technologies (Palo Alto, CA, USA).

### 2.2. Cell Culture and Treatment for the Induction of IR

Human hepatocarcinoma HepG2 and murine hepatocyte AML12 cell lines were obtained from the American Type Culture Collection (Manassas, VA, USA). HepG2 cells were cultured in Minimum Essential Medium (MEM; Welgene, Gyeongsan, Republic of Korea) supplemented with 10% fetal bovine serum (FBS; Welgene) and 1% antibiotic/antimycotic (A/A) solution containing 10,000 U/mL of penicillin G, 10 mg/mL of streptomycin, and 25 mg/mL of amphotericin B (Welgene). The culture medium was replaced with MEM containing 0.5% FBS without insulin to induce starvation in order to mimic IR conditions. After 24 h of incubation, the cells were treated with 30 mM glucose, 200 µM PA, and 1 nM insulin for an additional 24 h with or without tomatine at the specified concentrations. AML12 cells were cultured in Dulbecco’s Modified Eagle Medium/F-12 (DMEM/F12; Gibco, Carlsbad, CA, USA) supplemented with 10% FBS, 1% A/A solution, 1% insulin–transferrin–selenium solution (containing 10 µg/mL of insulin, 5.5 µg/mL of transferrin, and 5 ng/mL of selenium; Invitrogen, Carlsbad, CA, USA), and 40 ng/mL of dexamethasone (Sigma-Aldrich, St. Louis, MO, USA). The culture medium was replaced with DMEM/F12 containing 2% FBS without insulin for starvation to induce IR. Following 24 h of incubation, the cells were exposed to 27 mM glucose and 1 nM insulin for 24 h with or without tomatine at the specified concentrations, as previously described [16,17]. To inhibit AMPK activation, cells were pretreated with compound C (5 or 10 μM) for 24 h before tomatine treatment.

### 2.3. Cell Viability Assay

The water-soluble tetrazolium salt-1 (WST-1) assay (Roche Applied Science, Mannheim, Germany) was used to evaluate the cytotoxicity of tomatine. Briefly, the cells were seeded into 96-well plates at a density of 2 × 10^4^ cells/mL and treated with tomatine at the specified concentrations (0–1 µM). After 24 h of incubation, each well was added with 10% WST-1 solution prepared in the culture medium and then incubated for another 3 h at 37 °C in a 5% CO_2_ atmosphere. The cleavage of WST-1 to formazan by mitochondrial dehydrogenases was spectrophotometrically measured at 450 nm using a microplate reader (Molecular Devices, Sunnyvale, CA, USA). To further assess tomatine-induced cell death, the culture medium from treated cells was collected and centrifuged at 10,000× *g* for 5 min at 4 °C. The resulting supernatant was analyzed using an LDH assay kit (Roche Applied Science).

### 2.4. Glucose Consumption Assay

The glucose consumption rate was determined using the Glucose Colorimetric/Fluorometric Assay Kit (#CA-G005, eEnzyme, Gaithersburg, MD, USA). Briefly, the cell lysates (10 µg of protein equivalent) were diluted with the assay buffer, and the dilution was mixed with the same volume of the reaction buffer. The reaction mixtures were incubated for 10 min at 37 °C, and the glucose concentration was evaluated by fluorometric analysis (Ex/Em = 540/590) in a microplate reader (Molecular Devices) [18].

### 2.5. Quantitative Real-Time Polymerase Chain Reaction (RT-qPCR)

Total RNA was extracted using TRIzol Reagent (Invitrogen) in accordance with the manufacturer’s instructions. Next, the purified RNA was reverse-transcribed into complementary DNA (cDNA) using a cDNA synthesis kit (TOYOBO Co., Ltd., Osaka, Japan). Equal amounts of cDNA from each sample were then combined with the Prime SYBR™ Green PCR Master Mix (Roche Applied Science) for RT-qPCR. Subsequently, the reactions were performed on the CFX Connect™ Real-Time PCR Detection System (Bio-Rad, Hercules, CA, USA). Table S1 shows the primer sequences for the target genes. The relative mRNA expression levels were normalized to the expression of housekeeping genes (*Gapdh* or *Rplp0*) and quantified using the ΔΔCt method.

### 2.6. Western Blot Analysis

The cells were washed twice with cold phosphate-buffered saline (Welgene) and lysed using radioimmunoprecipitation assay buffer (#R0278; Sigma-Aldrich) supplemented with protease and proteasome inhibitors (Roche Applied Science, Indianapolis, IN, USA). The protein concentrations were determined using a bicinchoninic acid protein assay kit (#BCA1; Sigma-Aldrich). Equal amounts of protein were mixed with 5× sodium dodecyl sulfate–polyacrylamide gel electrophoresis (SDS-PAGE) buffer (Biosesang, Yongin-si, Republic of Korea), boiled at 95 °C for 5 min, and then cooled. The proteins from each sample were separated using 8% or 12% SDS-PAGE and transferred onto polyvinylidene fluoride membranes (Merck Millipore, Berlin, Germany). Then, the membranes were blocked with 5% nonfat dry milk in Tris-buffered saline with Tween-20 (TBST, Biosesang) for 1 h at room temperature. Subsequently, the membranes were incubated with the primary antibodies at 4 °C for 16 h and then washed with TBST for 1 h. Next, the membranes were incubated with peroxidase-conjugated secondary antibodies (Santa Cruz Biotechnology, Santa Cruz, CA, USA) for 1 h at room temperature. The protein bands were detected using a chemiluminescence detection system (Thermo Fisher Scientific, Sunnyvale, CA, USA), and densitometric analysis was performed using ImageJ software (version 1.54d).

### 2.7. Seahorse Real-Time Mitochondrial Respiration Analysis

The real-time oxygen consumption rate (OCR) was measured using an extracellular flux analyzer (Seahorse XF; Agilent Technologies). To achieve approximately 80% confluence, the cells were seeded in an XF cell culture plate (Agilent Technologies) at a density of 4 × 10^4^ cells/mL. The plate was incubated at 37 °C in a non-CO_2_ incubator for 1 h to allow degassing. The cells were then subjected to the Mito Stress Test using a nonbuffered basal medium (#103575-100, Agilent Technologies, XF DMEM, pH 7.4) in accordance with the manufacturer’s instructions. The mitochondrial OCR was measured at baseline and after sequential injections of oligomycin (Oligo, 1.5 µM), carbonyl cyanide 4-(trifluoromethoxy)phenylhydrazone (FCCP, 1.0 µM), and rotenone/antimycin A (1.0 µM). The OCR was recorded using an extracellular flux analyzer with three cycles of mixing (150 s), waiting (120 s), and measuring (210 s) after each injection. The mitochondrial respiration data were normalized to the control group. The total OCR and ATP production were calculated using the following equations: total OCR = OCR after FCCP treatment–nonmitochondrial OCR; ATP production = basal OCR−OCR after Oligo treatment. These calculations were performed as previously described [18].

### 2.8. Mitochondrial Isolation

Mitochondrial fractions were isolated from hepatocytes using the Mitochondria/Cytosol Fractionation Kit (#ab65320, Abcam) in accordance with the manufacturer’s instructions. Briefly, the cells were lysed in cytosol isolation buffer containing a protease inhibitor cocktail and 1 µM dithiothreitol. Afterward, they were disrupted using 70 strokes in a Dounce homogenizer. Cell debris and nuclear fractions were removed by centrifugation at 700× *g* for 10 min. Subsequently, the mitochondrial fractions were pelleted by centrifugation at 10,000× *g* for 25 min. For higher purity, the samples were washed with mitochondrial isolation buffer, and the final pellet obtained after centrifugation was used as the mitochondrial fraction.

### 2.9. Transfection

HepG2 and AML12 cell lines were transfected with either nontargeting siRNA (siCtrl, #sc-37007, Santa Cruz Biotechnology), human AMPK1/2-targeting siRNA (siAMPK, #sc-45312, Santa Cruz Biotechnology), or mouse siAMPK (#sc-45313, Santa Cruz Biotechnology) to knock down AMPK expression. siRNAs were transfected at a final concentration of 10 nM using Lipofectamine RNAiMAX (Invitrogen) at an siRNA-to-reagent ratio of 1:1.5. Following transfection, the cells were incubated at 37 °C for 24 h. The target protein knockdown efficiency was validated by Western blot analysis. Thereafter, the cells were processed for the designated experiments [18].

### 2.10. Statistical Analysis

All experiments were performed at least in triplicate. Data are expressed as the mean ± standard deviation (SD). Statistical differences were analyzed using one-way analysis of variance (ANOVA), followed by Tukey’s post hoc test for multiple comparisons or by unpaired two-tailed Student’s *t*-test. Statistical significance was considered at *p* < 0.05.

## 3. Results

### 3.1. Tomatine Improves Glucose Metabolism in IR Hepatocytes

**Figure 2:**
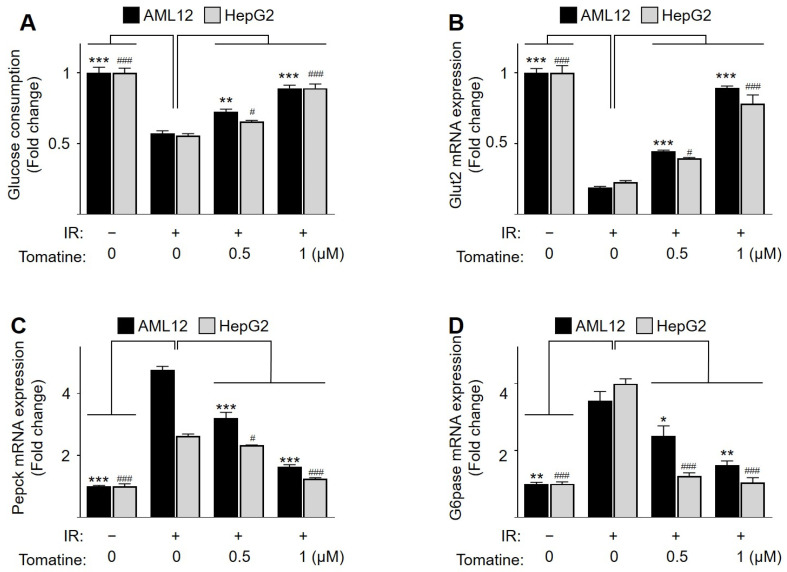
Effects of tomatine on glucose metabolism in IR hepatocytes. AML12 or HepG2 cells were cultured under conditions designed to induce IR, as described in Section 2. (**A**) Glucose consumption was measured, and (**B**–**D**) the mRNA expression levels of Glut2, Pepck, and G6pase were analyzed using RT-qPCR. Data are presented as the mean ± SD (n ≥ 3). Significant differences (*p* < 0.05) were determined using one-way ANOVA followed by Tukey’s post hoc test. *^,#^ *p* < 0.05, ** *p* < 0.001, and ***^,###^ *p* < 0.0001.

AML12 and HepG2 cells were treated with various concentrations of tomatine (0–1 µM) and assessed using WST-1 assay to evaluate the potential cytotoxicity of tomatine. Cells were treated with various concentrations of tomatine (0–1 µM), and no significant reduction in cell viability was observed at concentrations ≤1 µM (Figure 1B,C). Furthermore, tomatine treatment at these concentrations did not induce cell death, as evidenced by the lack of increased LDH release in either cell line (Supplementary Materials Figure S1). To investigate the effects of tomatine on glucose metabolism under hyperglycemic conditions, hepatocytes were starved and subsequently treated with a high-glucose/high-insulin solution to induce IR. Under these conditions, glucose consumption and Glut2 mRNA expression were significantly downregulated compared with the control group (Figure 2A,B). Additionally, the expression of gluconeogenesis-related genes, including phosphoenolpyruvate carboxykinase (*Pepck*) and glucose-6-phosphatase (*G6pase*), was upregulated (Figure 2C,D), confirming the successful establishment of the IR model. Interestingly, tomatine treatment increased glucose consumption and Glut2 mRNA expression in a dose-dependent manner in IR AML12 and HepG2 hepatocytes (Figure 2A,B). Moreover, tomatine dose-dependently restored the upregulated expression of *Pepck* and G6pase (Figure 2C,D). These results suggest that tomatine improves glucose metabolism in IR hepatocytes.

### 3.2. Tomatine Activates AMPK/PGC1a Signaling in IR Hepatocytes

**Figure 3:**
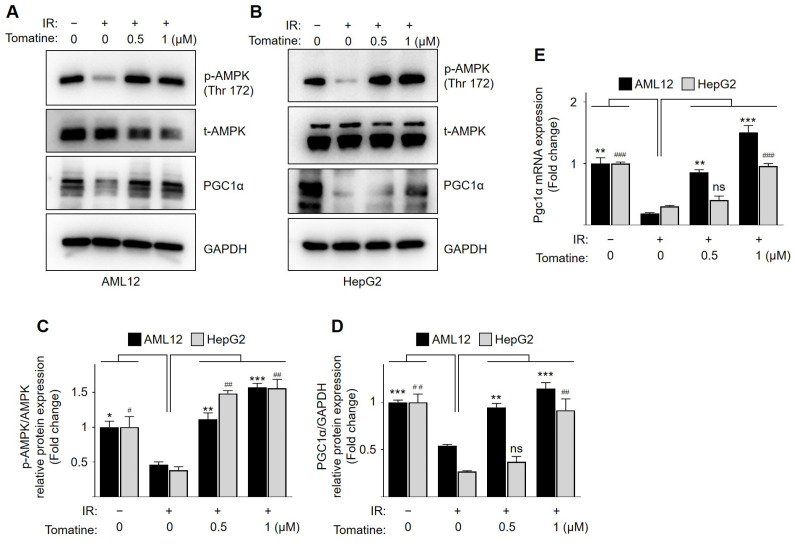
Effects of tomatine on AMPK/PGC1α signaling in IR hepatocytes. AML12 or HepG2 cells were cultured under conditions designed to induce IR, as described in Section 2. (**A**,**B**) Western blot analysis was performed for p-AMPK (Thr172), AMPK, PGC1α, and GAPDH. (**C**,**D**) The bar graphs represent the quantitative expression of p-AMPK (Thr172)/t-AMPK and PGC1α/GAPDH. (**E**) The mRNA expression of *Pgc1α* was analyzed using RT-qPCR. Data are presented as the mean ± SD (n ≥ 3). Significant differences (*p* < 0.05) were determined using one-way ANOVA followed by Tukey’s post hoc test. *^,#^ *p* < 0.05, **^,##^ *p* < 0.001, and ***^,###^ *p* < 0.0001. “ns” indicates nonsignificant differences.

AMPK is an important regulator of glucose metabolism as it maintains metabolic homeostasis in hepatocytes. To investigate the molecular mechanisms underlying the beneficial effects of tomatine on glucose metabolism in IR hepatocytes, we examined the activation of AMPK. Tomatine treatment dose-dependently upregulated the phosphorylation of AMPK at Thr172 in AML12 and HepG2 cells under normal culture conditions (Supplementary Materials Figure S2). Consistent with previous reports [19,20], the phosphorylation of AMPK and expression of PGC1α were significantly diminished under IR conditions compared with normal cells (Figure 3). However, tomatine treatment significantly and dose-dependently restored the phosphorylation of AMPK and expression of PGC1α in IR AML12 and HepG2 cells (Figure 3A–D). In addition, tomatine reversed the downregulated mRNA expression of PGC1α in IR conditions in a dose-dependent manner (Figure 3E). These findings suggest that tomatine acts as an AMPK activator, promoting the upregulation of PGC1α to improve hepatic glucose metabolism.

### 3.3. Tomatine Enhances Mitochondrial Oxidative Function in IR Hepatocytes

**Figure 4:**
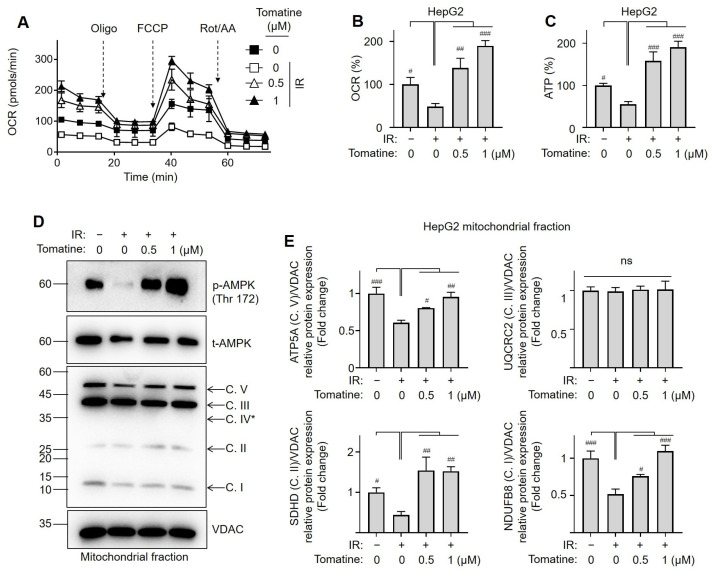
Effects of tomatine on mitochondrial oxidative function in IR hepatocytes. AML12 or HepG2 cells were cultured under conditions designed to induce IR, as described in Section 2. (**A**) The OCR was measured as described in Section 2. Representative data from three independent experiments are presented and normalized to cell numbers. (**B**) Total OCR was calculated and normalized to the protein content of each group. (**C**) The intracellular ATP levels were measured as described in Section 2. (**D**,**E**) The subunits of the OXPHOS complex were analyzed using SDS-PAGE followed by immunoblotting, with densitometric quantification provided. Data are shown as the mean ± SD (n = 3). Significant differences (*p* < 0.05) were determined using one-way ANOVA followed by Tukey’s post hoc test. ^#^ *p* < 0.05, ^##^ *p* < 0.001, and ^###^ *p* < 0.0001. “ns” indicates nonsignificant differences. * Complex IV subunit (with a theoretical molecular weight of 38 kDa) was not detected.

Considering the strong association between AMPK activation and mitochondrial function, we evaluated mitochondrial respiration and OXPHOS complex protein expression [21]. The OCR was significantly reduced under IR conditions compared with the control group (Figure 4A,B). Tomatine treatment dose-dependently restored the OCR suppressed by high glucose in IR HepG2 cells (Figure 4A,B and Supplementary Materials Figure S3). Furthermore, tomatine significantly and dose-dependently increased ATP production compared with the untreated controls (Figure 4C). In the mitochondrial fractions, the AMPK levels were significantly reduced but were restored by tomatine treatment in IR HepG2 cells (Figure 4D and Supplementary Materials Figure S4A). Although the expression of UQCRC2 (Complex III) remained unchanged, the levels of ATP5A (Complex V), SDHB (Complex II), and NDUFB8 (Complex I) were significantly downregulated compared with those in the control group (Figure 4D,E). Notably, tomatine treatment markedly upregulated the expression of these proteins under IR conditions (Figure 4D,E and Supplementary Materials Figure S4B). These results suggest that tomatine improves the mitochondrial quality by enhancing the oxidative capacity, thereby restoring the mitochondrial function in IR hepatocytes.

### 3.4. Tomatine Improves Glucose Metabolism in IR Hepatocytes by Activating AMPK

**Figure 5:**
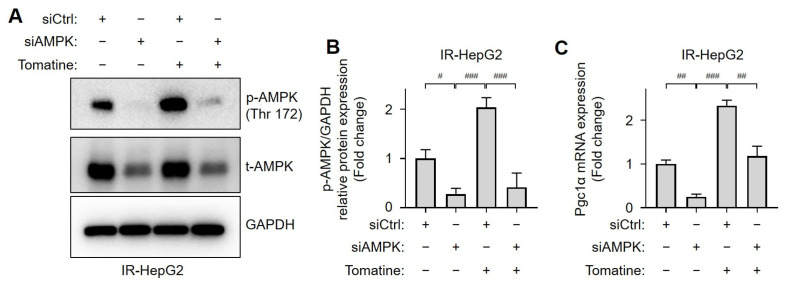
Effects of tomatine on the activation of AMPK in IR hepatocytes via an AMPK-dependent pathway. HepG2 cells were cultured under conditions designed to induce IR, as described in Section 2. The cells were transfected with either nontargeting siRNA (siCtrl) or AMPK-directed siRNA (siAMPK) for 24 h and then exposed to high glucose and insulin for 24 h, with or without tomatine (1 µM). (**A**) Western blot analysis was performed for p-AMPK (Thr172), AMPK, and GAPDH. (**B**) The quantitative bar graphs of p-AMPK (Thr172)/GAPDH are presented. (**C**) The mRNA expression of *Pgc1a* was analyzed using RT-qPCR. Data are presented as the mean ± SD (n ≥ 3). Significant differences (*p* < 0.05) were determined using one-way ANOVA followed by Tukey’s post hoc test. ^#^ *p* < 0.05, ^##^ *p* < 0.001, and ^###^ *p* < 0.0001.

**Figure 6:**
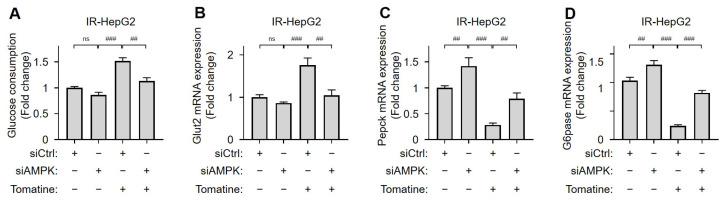
Effects of tomatine on glucose metabolism in IR hepatocytes via an AMPK-dependent pathway. HepG2 cells were cultured under conditions designed to induce IR, as described in Section 2. The cells were transfected with either nontargeting siRNA (siCtrl) or AMPK-directed siRNA (siAMPK) for 24 h and then exposed to high glucose and insulin for 24 h, with or without tomatine (1 µM). (**A**) The glucose consumption and (**B**–**D**) mRNA expression of *Glut2*, *Pepck*, and *G6pase* were analyzed using RT-qPCR. Data are shown as the mean ± SD (n ≥ 3). Significant differences (*p* < 0.05) were determined using one-way ANOVA followed by Tukey’s post hoc test. ^##^ *p* < 0.001, and ^###^ *p* < 0.0001. “ns” indicates nonsignificant differences.

AMPK was silenced using siRNA to investigate the role of AMPK activation in tomatine-treated IR hepatocytes. The successful downregulation of AMPK protein expression was confirmed in both IR hepatocyte cell lines (Figure 5A,B and Supplementary Materials Figure S5A). In AMPK-knockdown cells, the tomatine-induced increases in the phosphorylation of AMPK and mRNA expression of PGC1α were significantly reduced (Figure 5). Similarly, treatment with Compound C, an AMPK inhibitor, attenuated the tomatine-induced upregulation of PGC1α mRNA in IR HepG2 cells (Supplementary Materials Figure S5B). AMPK silencing did not affect glucose consumption or Glut2 mRNA expression in IR HepG2 cells. However, the expression of gluconeogenesis-related genes *Pepck* and *G6pase* was significantly increased (Figure 6). In IR HepG2 cells transfected with control siRNA, improvements in glucose metabolism induced by tomatine, including enhanced glucose consumption, upregulated Glut2 expression, and reduced expression of gluconeogenesis-related genes, were observed. Notably, AMPK-knockdown significantly reversed these improvements (Figure 6). Moreover, inhibition of AMPK with Compound C markedly reduced tomatine-induced glucose consumption in IR HepG2 cells (Supplementary Materials Figure S5C). These findings demonstrate that the activation of AMPK is essential for tomatine-induced improvements in glucose metabolism in IR hepatocytes.

### 3.5. Tomatine Improves Glucose Metabolism in IR Hepatocytes by Activating AMPK-Mediated Mitochondrial Oxidative Function

**Figure 7:**
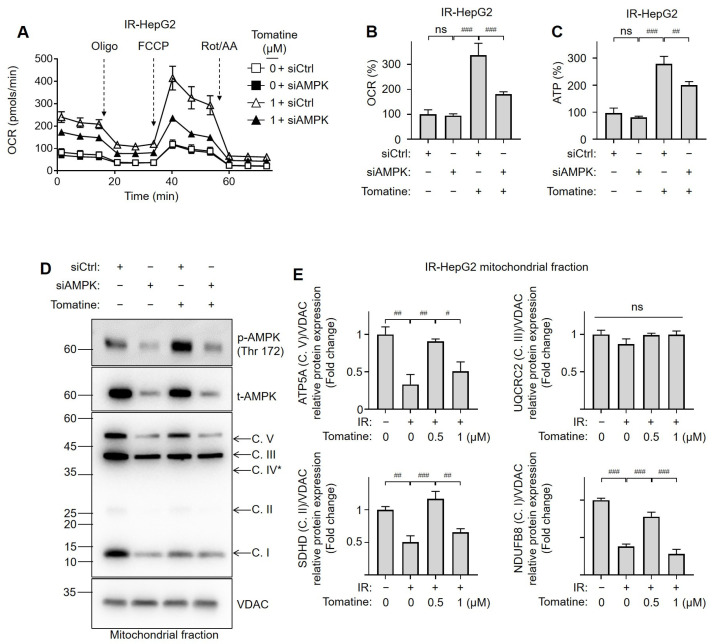
Effects of tomatine on mitochondrial oxidative function in IR hepatocytes via an AMPK-dependent pathway. AML12 or HepG2 cells were cultured under conditions designed to induce IR, as described in Section 2. The cells were transfected with either nontargeting siRNA (siCtrl) or AMPK-directed siRNA (siAMPK) for 24 h and then exposed to high glucose and insulin for 24 h, with or without tomatine (1 µM). (**A**) The OCR was measured as described in Section 2 and normalized to cell numbers. (**B**) Total OCR was calculated and normalized to the protein content of each group. (**C**) Intracellular ATP levels were measured. (**D**,**E**) The subunits of the OXPHOS complex were analyzed using SDS-PAGE followed by immunoblotting, with densitometric quantification provided. Data are shown as the mean ± SD (n ≥ 3). Significant differences (*p* < 0.05) were determined using one-way ANOVA followed by Tukey’s post hoc test. ^#^ *p* < 0.05, ^##^ *p* < 0.001, and ^###^ *p* < 0.0001. “ns” indicates nonsignificant differences. * Complex IV subunit (with a theoretical molecular weight of 38 kDa) was not detected.

Mitochondrial activity was assessed in AMPK-knockdown IR HepG2 cells to explore the role of mitochondrial function in the activation of AMPK during tomatine treatment. siAMPK transfection did not significantly alter the OCR or ATP levels in cells treated with DMSO (Figure 7A–C). However, the tomatine-induced increases in mitochondrial respiration and ATP production were significantly diminished in AMPK-knockdown cells (Figure 7A–C). We confirmed that siAMPK treatment effectively silenced the expression of AMPK in the mitochondrial fractions and that tomatine treatment increased the phosphorylation levels of AMPK (Figure 7D and Supplementary Materials Figure S6A). In addition, AMPK silencing substantially reduced the tomatine-induced upregulation of mitochondrial OXPHOS complex protein expression in IR HepG2 cells (Figure 7D,E and Supplementary Materials Figure S6B). These findings highlight the importance of AMPK-mediated mitochondrial respiration and protein quality in the beneficial effects of tomatine on glucose metabolism in IR hepatocytes.

## 4. Discussion

Glycoalkaloids (e.g., tomatine) exert distinct toxicological effects. The excessive intake of these compounds has been associated with gastrointestinal disturbances (e.g., vomiting and diarrhea), fever, and hypotension, primarily because of their inhibitory effects on acetylcholinesterase and butyrylcholinesterase [22]. The biological activity of glycoalkaloids is largely attributed to their ability to bind cholesterol and disrupt cellular membrane integrity through enzymatic inhibition [23]. Despite their potential cytotoxicity, several studies have highlighted their diverse bioactivities, including antiviral, antimicrobial, and immunomodulatory properties [24,25]. Notably, tomatine and its aglycone have attracted considerable attention for their therapeutic potential as multitargeted anticancer agents [14]. In the present study, we demonstrated that tomatine concentrations up to 1 µM did not induce cytotoxicity in AML12 and HepG2 hepatocytes. Furthermore, tomatine enhanced glucose metabolism in IR-induced hepatocytes by promoting glucose uptake and suppressing the expression of gluconeogenesis-related genes within nontoxic concentration ranges. These findings underscore the potential of tomatine as a therapeutic candidate for managing metabolic disorders, including IR.

AMPK plays a central role in regulating cellular energy by responding to shifts in energy status and stress [26]. It activates PGC-1α, an important regulator of mitochondrial biogenesis and function [27]. The activation of the AMPK/PGC-1α pathway increases mitochondrial protein expression and optimizes OXPHOS for efficient energy production [26,27]. However, this pathway is disrupted by IR, impairing glucose oxidation, reducing ATP production, and diminishing the oxidative capacity [28]. This creates a negative feedback loop that further suppresses the activity of AMPK, exacerbating mitochondrial dysfunction, and contributing to metabolic abnormalities (e.g., hyperglycemia) [28]. Moreover, activation of the AMPK/PGC-1α pathway promotes interactions with mitochondrial proteins that enhance mitochondrial quality by improving mitophagy (the removal of damaged mitochondria) and maintaining balanced mitochondrial dynamics (fusion and fission)—processes closely linked to the progression of various metabolic diseases [29]. Patients with T2DM often exhibit reduced mitochondrial content and functionality, indicating impaired mitochondrial biogenesis [30]. Similarly, individuals with fatty liver disease frequently show diminished mitochondrial respiration and ATP production in IR hepatocytes [31]. These findings underscore the therapeutic potential of strategies aimed at enhancing mitochondrial oxidative function to alleviate IR. In the present study, tomatine treatment restored AMPK/PGC-1α protein expression in IR AML12 and HepG2 cells. Consistent with previous reports, IR-induced hepatocytes exhibited downregulated mitochondrial OXPHOS complex proteins (Complexes I, II, and IV), which were significantly upregulated following tomatine treatment. Additionally, tomatine enhanced mitochondrial respiration in IR HepG2 cells, an effect that was notably diminished by both genetic and chemical inhibition of AMPK. Importantly, AMPK inhibition also impaired tomatine-mediated improvements in glucose uptake, underscoring the critical role of the AMPK/PGC-1α pathway in mediating the anti-IR effects of tomatine.

The phosphatidylinositol-3-kinase (PI3K)/protein kinase B (Akt) pathway is pivotal for regulating hepatic glucose metabolism, a process modulated by insulin receptor substrate (IRS)-1 [32]. Phosphorylated Akt (p-Akt) inhibits glycogen synthase kinase-3β, thereby reducing the phosphorylation of glycogen synthase and promoting the synthesis of glycogen [33]. Moreover, p-Akt suppresses key gluconeogenic enzymes, including Pepck and G6pase [34]. The PI3K/Akt pathway also facilitates glucose transport by upregulating glucose transporters, such as GLUT2, in hepatocytes [35]. Thus, the dysregulation of the IRS/PI3K/Akt axis significantly contributes to hepatic IR. A previous study suggested that the activation of AMPK can stimulate the PI3K/Akt pathway, improving insulin sensitivity and supporting energy homeostasis [28]. AMPK also enhances the phosphorylation and expression of IRS-1, further regulating this pathway [11]. Various studies have identified compounds that are capable of activating AMPK to modulate the IRS/PI3K/Akt axis [36]. For example, the natural flavonoid chrysin has been shown to activate the AMPK/PI3K/Akt pathway, improving glucose metabolism in IR HepG2 cells [37]. Similarly, metformin restores glucose uptake by activating the Akt-stimulated glucose transporter system through the phosphorylation of AMPK at Thr 172 [38]. Other studies indicate that mitochondrial peptides and oleuropein alleviate type 2 diabetes mellitus (T2DM) symptoms by activating AMPK [39,40]. In this study, tomatine treatment restored Glut2 expression in IR hepatocytes and significantly reduced the upregulation of gluconeogenic enzymes such as Pepck and G6pase. However, these beneficial effects were significantly reversed when AMPK was inhibited, highlighting AMPK’s critical role in tomatine’s mechanism of action. Typically, AMPK phosphorylation at Thr172 is mediated by calmodulin-dependent protein kinase II (CAMKII) and liver kinase B1 in response to cellular stress, such as elevated intracellular Ca^2+^ levels and decreased ATP/ADP ratios [41]. Interestingly, the steroidal alkaloid tomatidine has been reported to activate CAMKII, leading to increased AMPK phosphorylation in HepG2 cells and the subsequent suppression of lipid accumulation [42]. Therefore, further studies are needed to elucidate the precise molecular mechanisms underlying the antidiabetic effects of tomatine and its potential therapeutic applications in metabolic disorders.

The present study explored the effects of tomatine on glucose metabolism using high-glucose-induced HepG2 and AML12 cells. However, several limitations should be addressed to strengthen our findings. Although mitochondrial energy metabolism is closely linked to the AMPK pathway, further experiments targeting insulin receptor-mediated signaling are necessary to fully elucidate the underlying molecular mechanisms. Additionally, employing primary hepatocytes or human iPSC-derived liver cells could provide more physiologically relevant insights into the improvement of IR. In vivo studies are also essential to evaluate the therapeutic efficacy and safety of tomatine for enhancing glucose metabolism in IR hepatocytes. Overall, our findings highlight the potential of tomatine as a therapeutic agent for managing metabolic disorders such as T2DM. Nonetheless, further studies are needed to confirm its safety and efficacy in vivo and to determine its suitability for clinical applications.

## 5. Conclusions

Tomatine is nontoxic and effectively improves glucose metabolism in IR-induced hepatocytes by enhancing glucose uptake, suppressing gluconeogenesis, and promoting mitochondrial oxidative function. In addition, tomatine treatment significantly activates the AMPK/PGC-1α signaling pathway, and its metabolic benefits are AMPK-dependent. These findings highlight the potential of tomatine as a novel preventive and therapeutic option for metabolic disorders, underscoring the need for further in vivo and clinical studies to validate its efficacy and safety.

## Data Availability

The data presented in this study are available upon request from the corresponding author.

## Supplementary Materials

The following supporting information can be downloaded at: https://www.mdpi.com/article/10.3390/cells14050329/s1, Table S1. Oligonucleotide sequences used for RT-qPCR analysis; Figure S1. Cytotoxicity of tomatine in hepatocytes; Figure S2. Tomatine dose-dependently increases the phosphorylation of AMPK in hepatocytes; Figure S3. Effects of tomatine on mitochondrial oxidative function in IR hepatocytes; Figure S4. Effects of tomatine on mitochondrial oxidative function in IR hepatocytes; Figure S5. Tomatine activates AMPK/PGC1α signaling in IR hepatocytes by activating AMPK; Figure S6. Effects of tomatine on mitochondrial oxidative function in IR hepatocytes via an AMPK-dependent pathway.
