# Supplementary material for: Tomatine Improves Glucose Metabolism and Mitochondrial Respiration in Insulin-Resistant Hepatocyte Cell Lines AML12 and HepG2 via an AMP-Activated Protein Kinase-Dependent Pathway
Source: Cells. 2025 Feb 23;14(5):329. doi: 10.3390/cells14050329 (PMC11898437; doi:10.3390/cells14050329)
Supplement: Supplementary file 1 [file cells-14-00329-s001.zip › Supplementary Figures_2nd revised.pdf]

Supplementary Figures

Tomatine Improves Glucose Metabolism and Mitochondrial Respiration in Insulin-Resistant Hepatocyte Cell Lines AML12 and HepG2 via an AMP-Activated Protein Kinase-Dependent Pathway

Yu Geon Lee [1] and Donghwan Kim [1,*]

[1] Food Functionality Research Division, Korea Food Research Institute (KFRI), Wanju-gun, Jeonbuk-do 55365, Republic of Korea; ugun2@kfri.re.kr (Y.G.L.); kimd@kfri.re.kr (D.K.)

*Correspondence: kimd@kfri.re.kr; Tel.: +82-63-219-9586

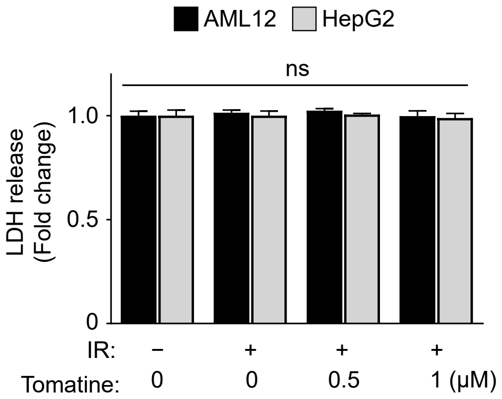

Supplementary Figure S1. Cytotoxicity of tomatine in hepatocytes.
AML12 or HepG2 cells were treated for 24 h with various concentrations (0–1 μM) of tomatine. Cells were treated with tomatine, and then the media were collected and analyzed by LDH assay kit.

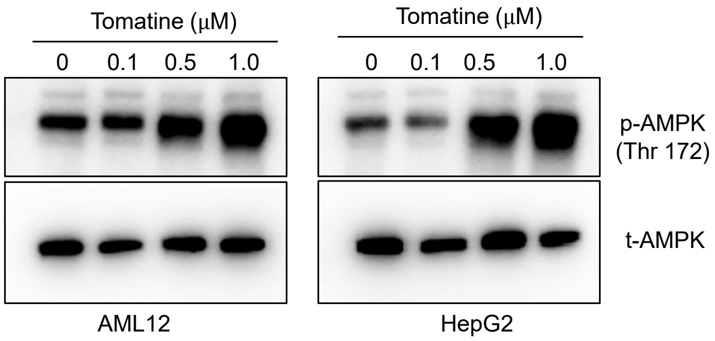

Supplementary Figure S2. Tomatine dose-dependently increases the phosphorylation of AMPK in hepatocytes.

AML12 or HepG2 cells were cultured in complete medium for 24 h and then exposed to tomatine (0–1 μM). (A and B) Western blot analysis was performed for p-AMPK (Thr172) and AMPK.

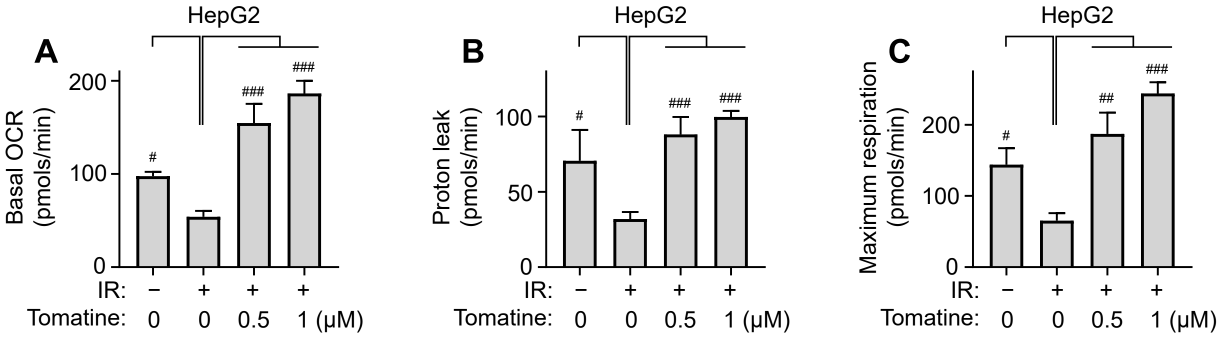

Supplementary Figure S3. Effects of tomatine on mitochondrial oxidative function in IR hepatocytes.

HepG2 cells were cultured under conditions designed to induce IR, as described in Section 2. (A–C) The OCR was measured as described in Section 2. Data are shown as the mean ± SD (n = 3). Significant differences ($p < 0.05$) were determined using one-way ANOVA followed by Tukey's post hoc test. $^{\#}p < 0.05$, $^{\#\#}p < 0.001$, and $^{\#\#\#}p < 0.0001$.

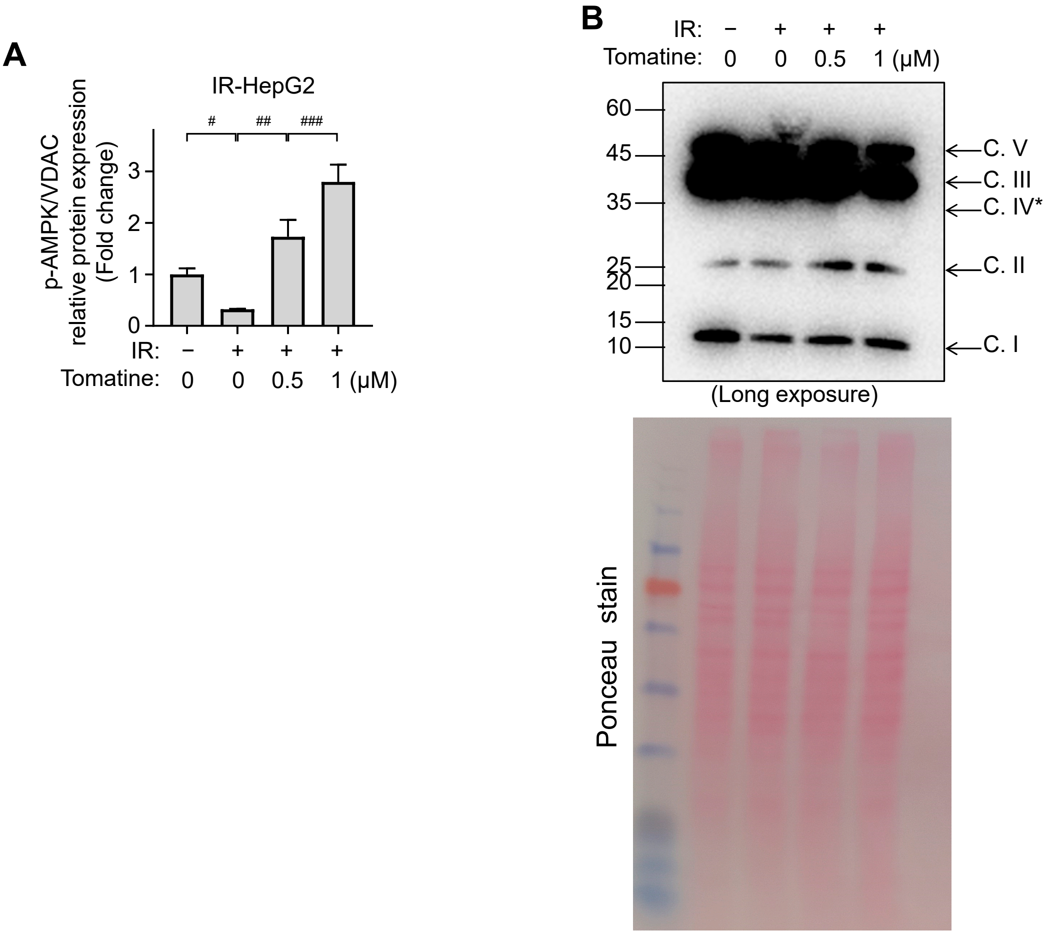

Supplementary Figure S4. Effects of tomatine on mitochondrial oxidative function in IR hepatocytes.

AML12 or HepG2 cells were cultured under conditions designed to induce IR, as described in Section 2. (A) Western blot analysis of the purified mitochondrial fractions was performed for for p-AMPK (Thr172), AMPK, and VDAC. The quantitative bar graphs of p-AMPK (Thr172)/VDAC are presented. (B) The subunits of the OXPHOS complex were analyzed using SDS-PAGE followed by immunoblotting. The Ponceau stain of the PVDF membrane is shown to confirm equal protein loading. Data are shown as the mean ± SD (n = 3). Significant differences ($p < 0.05$) were determined using one-way ANOVA followed by Tukey's post hoc test. $^{\#}p < 0.05$, $^{\#\#}p < 0.001$, and $^{\#\#\#}p < 0.0001$. *The Complex IV subunit (with a theoretical molecular weight of 38 kDa) was not detected.

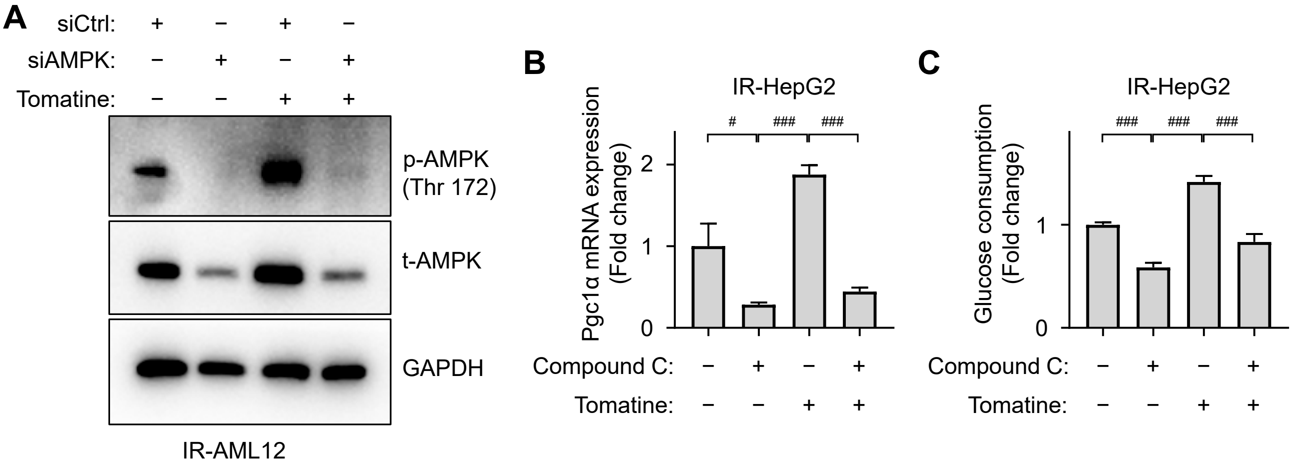

Supplementary Figure S5. Tomatine activates AMPK/PGC1α signaling in IR hepatocytes by activating AMPK.

AML12 or HepG2 cells were cultured under conditions designed to induce IR, as described in Section 2. (A) Western blot analysis was performed for p-AMPK (Thr172), AMPK, and GAPDH. (B) The mRNA expression of *Pgc1a* was analyzed using RT-qPCR. (C) Glucose consumption was measured, Data are presented as the mean ± SD (n ≥ 3). Significant differences ($p < 0.05$) were determined using one-way ANOVA followed by Tukey's post hoc test.`

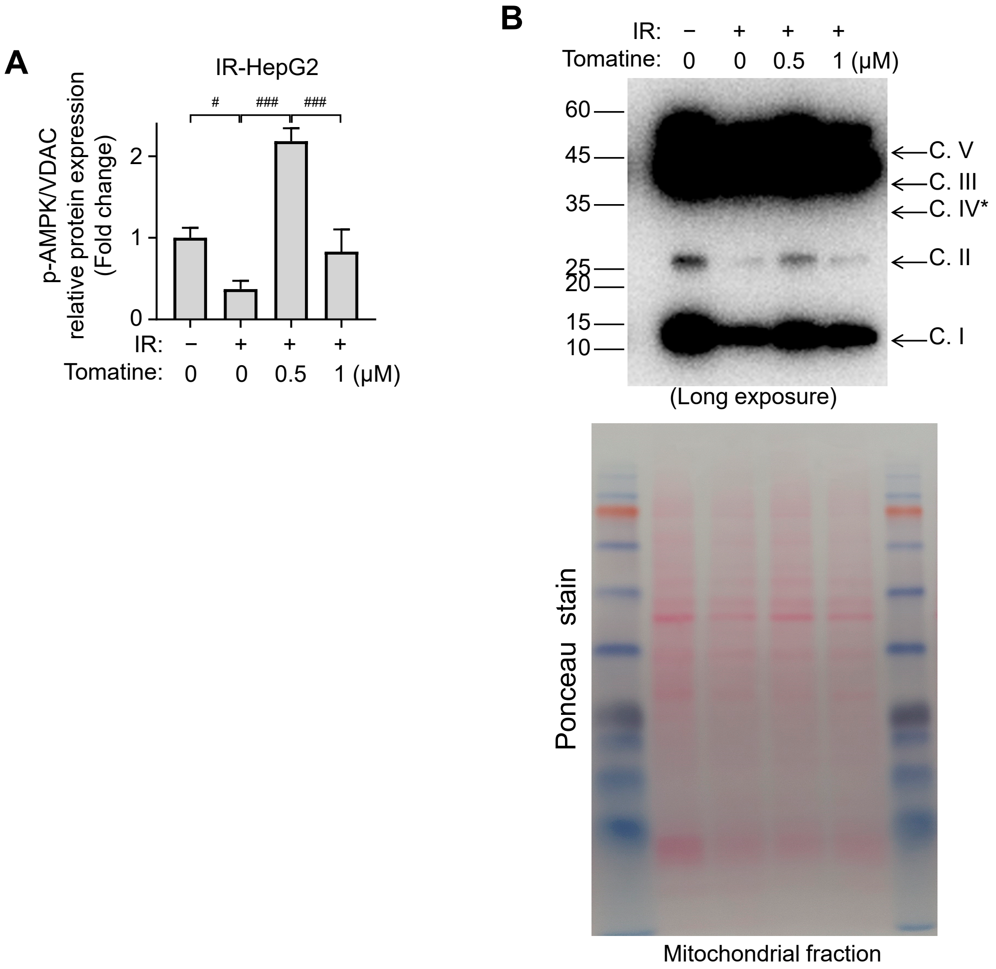

Supplementary Figure S6. Effects of tomatine on mitochondrial oxidative function in IR hepatocytes via an AMPK-dependent pathway.

AML12 or HepG2 cells were cultured under conditions designed to induce IR, as described in Section 2. The cells were transfected with either nontargeting siRNA (siCtrl) or AMPK-directed siRNA (siAMPK) for 24 h and then exposed to high glucose and insulin for 24 h, with or without tomatine (1 μM). (A) Western blot analysis of the purified mitochondrial fractions was performed for p-AMPK (Thr172), AMPK, and VDAC. The quantitative bar graphs of p-AMPK (Thr172)/VDAC are presented. (B) The subunits of the OXPHOS complex were analyzed using SDS-PAGE followed by immunoblotting. The Ponceau stain of the PVDF membrane is shown to confirm equal protein loading. Data are shown as the mean ± SD (n = 3). Significant differences ($p < 0.05$) were determined using one-way ANOVA followed by Tukey's post hoc test. $^{\#}p < 0.05$, $^{\#\#}p < 0.001$, and $^{\#\#\#}p < 0.0001$. *The Complex IV subunit (with a theoretical molecular weight of 38 kDa) was not detected.
