# Supplementary material for: Tomatine Improves Glucose Metabolism and Mitochondrial Respiration in Insulin-Resistant Hepatocyte Cell Lines AML12 and HepG2 via an AMP-Activated Protein Kinase-Dependent Pathway
Source: Cells. 2025 Feb 23;14(5):329. doi: 10.3390/cells14050329 (PMC11898437; doi:10.3390/cells14050329)
Supplement: Supplementary file 1 [file cells-14-00329-s001.zip › Supplementary Table_revised.pdf]

Supplementary Table S1. Oligonucleotide sequences used for RT-qPCR analysis.

Target gene	Direction	Sequence (5′-3′)
Mouse Glut2	forward	GTGTCTGCTACTGCTCTTCTGTC
	reverse	GACATCCTCAGTTCCTCTTAGTCTC
Mouse Pepck	forward	AAGCATTCAACGCCAGGTTC
	reverse	GGGCGAGTCTGTCAGTTCAAT
Mouse G6pase	forward	GGACACCGACTACTACAGCAACAG
	reverse	GCATGGCCAGAGGGACTTC
Mouse Pgc1α	forward	AATGAGGGCAATCCGTCTTCA
	reverse	AAGTGGTGTAGCGACCAATCG
Human Glut2	forward	GCTGCTCAACTAATCACCATGC
	reverse	TGGTCCCAATTTTGAAAACCCC
Human Pepck	forward	CTTTGGAGGCCGTAGACCTG
	reverse	GCCTTTATGTTCTGCAGCCG
Human G6pase	forward	CATTGACACCACACCCTTTGC
	reverse	CCCTGTACATGCTGGAGTTGAG
Human Pgc1α	forward	TCTGAGTCTGTATGGAGTGACAT
	reverse	CCAAGTCGTTCACATCTAGTTCA
Rplp0	forward	GTGCTGATGGGCAAGAAC
	reverse	AGGTCCTCCTTGGTGAAC

Note: Glucose Transporter Type 2, *Glut2*; Phosphoenolpyruvate Carboxykinas e, *Pepck*; Glucose-6-Phosphatase, *G6pase*; Peroxisome proliferator-activated rece ptor γ coactivator 1 α, Pgc1α; Ribosomal protein lateral stalk subunit P0, Rpl p0.
